# Magnetic Iron Oxide Nanoparticles: Synthesis, Characterization and Functionalization for Biomedical Applications in the Central Nervous System

**DOI:** 10.3390/ma12030465

**Published:** 2019-02-02

**Authors:** Shoeb Anwar Mohammed Khawja Ansari, Eleonora Ficiarà, Federico Alessandro Ruffinatti, Ilaria Stura, Monica Argenziano, Ornella Abollino, Roberta Cavalli, Caterina Guiot, Federico D’Agata

**Affiliations:** 1 Department of Neuroscience, University of Turin, 10124 Turin, Italy; shoebanwarmohammedkhawja.ansari@unito.it (S.A.M.K.A.); eleonora.ficiara@unito.it (E.F.); caterina.guiot@unito.it (C.G.); 2 Department of Drug Sciences, University of Eastern Piedmont Amedeo Avogadro, 28100 Novara, Italy; federicoalessandro.ruffinatti@uniupo.it; 3 Department of Public Health and Pediatrics, University of Turin, 10124 Turin, Italy; ilaria.stura@unito.it; 4 Department of Drug Science and Technology, University of Turin, 10124 Turin, Italy; monica.argenziano@unito.it (M.A.); roberta.cavalli@unito.it (R.C.); 5 Department of Chemistry, University of Turin, 10124 Turin, Italy; ornella.abollino@unito.it

**Keywords:** iron oxide nanoparticles, magnetic nanoparticles, synthesis, biomedical applications, central nervous system

## Abstract

Magnetic Nanoparticles (MNPs) are of great interest in biomedicine, due to their wide range of applications. During recent years, one of the most challenging goals is the development of new strategies to finely tune the unique properties of MNPs, in order to improve their effectiveness in the biomedical field. This review provides an up-to-date overview of the methods of synthesis and functionalization of MNPs focusing on Iron Oxide Nanoparticles (IONPs). Firstly, synthesis strategies for fabricating IONPs of different composition, sizes, shapes, and structures are outlined. We describe the close link between physicochemical properties and magnetic characterization, essential to developing innovative and powerful magnetic-driven nanocarriers. In conclusion, we provide a complete background of IONPs functionalization, safety, and applications for the treatment of Central Nervous System disorders.

## 1. Introduction

In the last decades, the power of nanotechnology in numerous fields including biomedical sciences has been exploited. Nanoparticles (NPs) are solid colloidal particles ranging in size from 1 to 100 nm. Due to their dimensions comparable with those of cells, viruses, proteins, and genes, they opened the potentiality of interacting with fundamental biological processes [1]. In recent years, much attention has been paid to the synthesis of a different kind of NPs as nano-medical materials. Among them, engineered Magnetic Nanoparticles (MNPs) made of iron, cobalt, or nickel oxides exhibit special properties, including high surface-to-volume ratio and high magnetic moment, allowing potential manipulation by an external magnetic field [2]. Especially, MNPs manufactured with ferromagnetic material, i.e., Iron Oxide Nanoparticles (IONPs), made of magnetite (Fe_3_O_4_) and maghemite (γ-Fe_2_O_3_) combine ideal biocompatibility with superparamagnetic properties allowing widespread biomedical uses such as targeted drug delivery, bioimaging, hyperthermia, photoablation therapy, biosensors, and theranostics applications [3,4].

The enhancement of effective drug delivery by magnetic driving of sensitive MNPs has been especially explored to treat diseases of the Central Nervous System (CNS). In fact, due to the aging of the population, CNS pathologies such as neurodegenerative diseases are increasingly becoming a relevant medical and social issue [5]. Current pharmacological treatments are mainly based on the systemic delivery of active substances into the CNS, whose effectiveness is seriously limited due to the presence of blood-brain-barrier (BBB). MNPs are therefore considered powerful tools to cross BBB by means of physical mechanisms and properly deliver the drug cargo in the brain. There are many in vitro and in vivo evidences of BBB trespassing by MNPs under magnetic fields to deliver therapeutic agents in the CNS [6,7].

Several steps have to be taken into account to design MNPs suitable for magnetic drug delivery in CNS. Currently, many methods of synthesis are available making it possible to produce a wide spectrum of different MNPs, by optimizing protocols, in order to obtain the best physicochemical properties [8]. Focusing on IONPs, they are usually made of a crystalline core and a surface coating (e.g., dextran, citrate, chitosan, polyethylene glycol, albumin, etc.), whose dimensions are tunable to improve their stability, enhance their biocompatibility, and optimize their bio-distribution. NP coating may be modified with specific targeting molecules or drugs. This variety of physical features and coatings characterizes IONP action mechanisms and toxicity patterns [9]. Moreover, since the synthesis methods affect the IONP size distribution, the degree of structural defects, the surface chemistry, and the magnetic behavior, they finally also determine the interaction with biological barriers—such as BBB and lipid bilayer of a cell membrane—and the consequent biocompatibility in the living organisms [10,11]. Furthermore, the iron content in IONPs has a key role in the general iron homeostasis of the body and in the CNS environment, highly sensitive to iron imbalance, especially in neurodegenerative diseases [12].

This review focuses on the recent developments of IONP synthesis methods and functionalization, highlighting the links between IONP physical characteristics and magnetic properties. Finally, biomedical applications, safety, and toxicity of IONPs in CNS are also briefly outlined.

## 2. Methods

PRISMA guidelines were followed for the collection and selection of the reported papers (http://prisma-statement.org). PubMed and ScienceDirect were used as scientific reference resources. Initially, searches were limited to the articles published in English in the last 2 years and up to December 2018, mainly because of the fast obsolescence of the literature related to this rapidly evolving scientific field. Combinations of the following keywords were used: Iron oxide, magnetic, nanoparticles, synthesis, properties, magnetization, functionalization, central nervous system, and toxicity. The logical combination of the keywords used for the query has been:

Iron oxide AND magnetic AND nanoparticles AND synthesis AND central nervous system
OR
Iron oxide AND nanoparticles AND magnetization AND properties AND central nervous system
OR
Iron oxide AND nanoparticles AND functionalization AND central nervous system
OR
Iron oxide AND nanoparticles AND toxicity AND central nervous system.

After the removal of duplicates from query results, relevant papers were chosen by reading titles and abstract content, based on the following criteria: Description of synthesis methods and functionalization of IONPs; possibly containing data on magnetic characterization; possibly containing data about the toxicity on CNS target cells (neurons or glial cells). In general, only those papers concerning CNS biomedical applications of iron oxide NPs, possibly steered by magnetic force, were retained for subsequent discussion. On the contrary, papers describing MNPs made of different materials or addressing the issue of targeting body districts different from CNS, were generally discarded. Finally, the so filtered papers—comprised of both original research articles and reviews—were integrally read and their bibliography was manually screened to additionally retrieve the largest number of seminal works upon which the recent and most relevant literature is founded.

## 3. Results

Overall, nine hundred and fifty-one (951) papers were collected through database searching. The second step of bibliography synthesis consisted in the manual selection of a subset of the so collected papers, essentially on the basis of their abstract and their actual contents (in particular the presence of CNS-specific details about IONP behavior and their magnetic characterization). The number of works resulting from this filtering procedure was fifty-three (53). Sixty-three (63) further references were then added—as stated in Methods Section—by manually screening the bibliography of the collected publications, for a final number of one hundred and sixteen (116) entries. As a result of this last step, more than half (54%) of our reported references are prior to 2017. In particular, apart from the last two years, also the period ranging between 2012 and 2015 is highly represented in this review, having thirty-eight (38) cited works. 

The selected literature was classified into four main groups:

1. Methods of synthesis of IONPs [13,14,15,16,17,18,19,20,21,22,23,24,25,26,27,28,29,30,31,32,33,34,35,36,37,38,39,40,41,42,43,44,45,46,47,48,49,50,51,52,53,54,55,56]
2. Characterization of IONPs [57,58,59,60,61,62,63,64,65,66,67,68,69,70,71,72,73,74,75,76,77,78,79,80,81,82,83,84,85]
3. Functionalization of IONPs for biomedical CNS applications [86,87,88,89,90,91,92,93,94,95,96,97,98,99,100,101,102,103,104,105]
4. Toxicity of IONPs in CNS [106,107,108,109,110,111,112,113,114,115,116].

## 4. Discussion

### 4.1. Synthesis of IONPs

**Figure 1:**
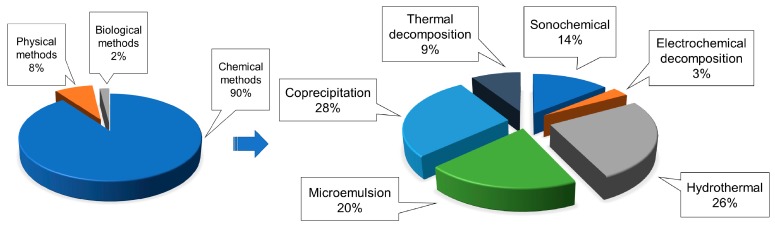
A comparison among the prevalence of the main methods currently existing for the synthesis of IONPs, with the detailed chemical methods prevalence. Modified from [14].

The most commonly used materials for the synthesis of MNPs consist of compounds (usually oxides) of iron, cobalt, or nickel, in combination with other metals such as copper, zinc, strontium, and barium. However, as already mentioned, this review focuses only on those procedures and precursor compounds suitable for the production of iron oxide MNPs. In general, the synthesis of MNPs is a very critical multistep procedure, which must be optimized since its early design phase, given that even a small variation in the production process can significantly change the desired outcome [8]. For this reason, both physical and chemical properties of the particles needs to be strictly controlled in order to fit a number of different applications [13]. Specifically, depending on the desired features and on the final field of application, three main routes for IONP preparation have been developed during last decades: Chemical, physical, and biological, the former comprising about the 90% of all synthesis methods (Figure 1) [14,15].

#### 4.1.1. Co-Precipitation Method 

**Figure 2:**
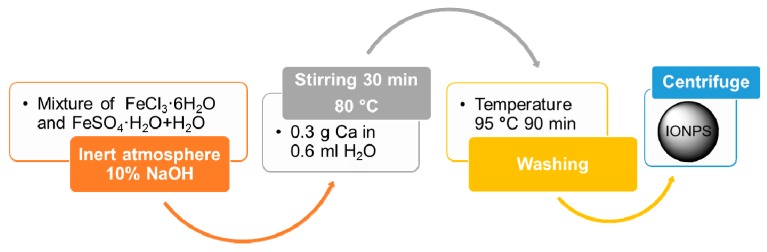
Flowchart for the synthesis of IONPs by co-precipitation [19]. In this example, Fe_3_O_4_ NPs are prepared from a mixture of FeSO_4_ and FeCl_3_ in the molar ratio of 1:2. Magnetic phase and particle size can be adjusted by varying Fe^2+^/Fe^3+^ ratio, temperature, pH, and the type of base used.

The co-precipitation method is probably the most popular method for IONP synthesis. In particular, it is widely used in biomedical applications, because of the non-toxic nature of the materials usually employed [16]. The term “co-precipitation” refers to the phenomenon by which a precipitate can carry down one or more substance—normally soluble in those particular conditions—through nucleation and grain growth. This method allows MNPs to be synthesized in an inert nitrogen atmosphere at room temperature [17]. For a schematic representation of such a synthesis technique, see Figure 2, where a typical procedure for the production of spherical IONPs is shown [18]. Through the co-precipitation synthesis, it is possible to obtain MNPs broadly distributed around a mean diameter value ranging from 5 to 40 nm (Table 1). More generally, size, shape, and magnetic properties of the resulting IONPs depend on reaction conditions such as the type of salts used [19], pH, and ionic strength [20,21]. 

#### 4.1.2. Thermal Decomposition Method

Thermal decomposition is one of the most effective methods to produce narrow size distribution MNPs, also allowing for the fine-tuning of particle mean diameter [22]. In particular, thermal decomposition can be achieved by two different protocols, namely “heating-up” and “hot-injection”. The heating-up process implies the continuous heating of a pre-mixed solution of precursor compounds, surfactants, and solvent, up to a given temperature at which NPs start clustering and growing [23,24]. On the contrary, the hot-injection method induces a fast and homogeneous nucleation by injecting reagents into a hot surfactant solution followed by a controlled growth phase. In any case, both the processes are based on the same principle consisting in heating a non-magnetic organometallic precursor compound in the presence of organic solvents and surfactants [25]. Usually, iron carbonyls and acetylacetonates are used as non-magnetic precursors, while fatty acids, rather than oleic acid, are commonly used as surfactants [18]. Importantly, argon plays an important role to maintain the atmosphere inert. The optimal temperature required for this reaction ranges between 100 °C and 350 °C, leading to the production of crystalline MNPs sized between 4 and 30 nm in diameter (Table 1) and exhibiting a high degree of uniformity (i.e., narrow size distributions) [22,26]. In this context, temperature and time of reaction are important factors to control particle size.

#### 4.1.3. Microemulsion Method

**Figure 3:**
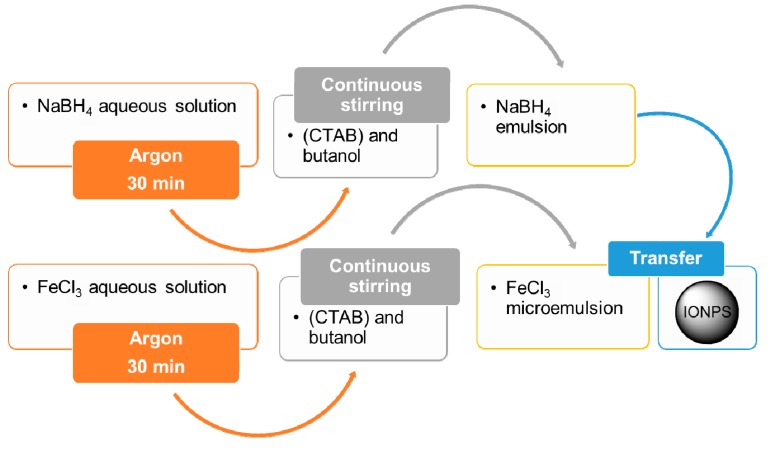
Flowchart for the synthesis of IONPs by microemulsion. Two W/O microemulsions (respectively with FeCl_3_ and NaBH_4_ in aqueous solution) are used for the preparation of MNPs with an iron core coated by a Fe_3_O_4_ shell [28]. Abbreviation: CTAB = cetyltrimethylammonium bromide.

The microemulsion is a thermodynamically stable dispersion of two immiscible liquids in the presence of a surfactant, which forms a monolayer at the interface between oil and water, possibly exhibiting an ultralow interfacial tension [27]. In microemulsion, IONPs are typically synthetized by intramicellar nucleation and growth, following the standard procedure exemplified in Figure 3 [28]. The physicochemical properties of NPs prepared by such a technique essentially depend upon the choice of the surfactant. Specifically, nanoparticles result in spherical shape, nearly monodispersed, with an average diameter ranging between 10 and 25 nm [28,29]. In this context, water-in-Oil (W/O) microemulsions are called reverse micelles [30].

#### 4.1.4. Hydrothermal Method

A broad range of crystalline IONPs can be synthetized by using the hydrothermal method. The general system consists of (solid) metal linoleate, an ethanol-linoleic acid liquid phase, and water-ethanol solution kept under hydrothermal (i.e., high-temperature and high-pressure) conditions [31]. Specifically, the typical reaction temperature to perform hydrothermal synthesis is around 220 °C, while the required pressure is above 10^7^ Pa, for a total reaction time of about 72 h [18,31,32]. Usually, a temperature gradient is created within a Teflon-lined stainless-steel autoclave whose cooler end will host the deposition of the mineral solute, finally growing the desired crystal. Through this technique, shape and size of the resulting NPs are generally very uniform, with the possibility of tuning NP size from few nanometers to several hundred (see Table 1) [31,33,34]. However, in order for the magnetic properties to be effective, the most interesting diameters are the smallest ones, since the upper limit for the formation of single domain particles is about 80 nm [18]. In general, particle size and size distribution depend upon the precursor concentration, total reaction time, and the temperature at which the reaction takes place [35]. Moreover, the hydrothermal synthesis is eco-friendly and versatile as no organic solvents or post-treatments are required [36].

#### 4.1.5. Polyol Method

**Figure 4:**
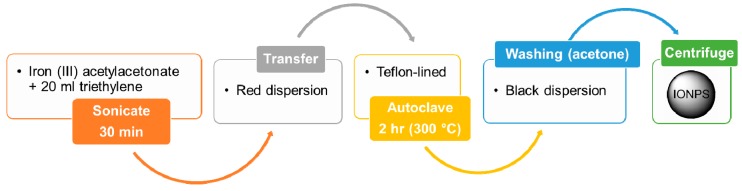
Flowchart for the synthesis of IONPs by polyol method [38]. IONPs are here prepared from a reaction between a metal acetylacetonate (precursor compound) and triethylene (diol solvent).

The polyol method allows synthetizing uniform MNPs at a relatively low temperature and it is based on precursor compounds such as oxides, acetates and nitrates dissolved or suspended in diols (Figure 4). It is a versatile and up-scalable method suitable for the production of large batches of IONPs, encompassing a wide range of possible size, from ultra-small spheres of 4 nm in diameter to larger ones up to 100 nm, depending on reaction conditions (e.g., temperature, reaction time, heating profile, nature of the polyol solvent, or organometallic precursors) [37,38]. The polyol method is also a useful technique for the synthesis of nanocrystalline alloys and bio-metallic clusters. Table 1 shows the typical time and temperature required to obtain NPs through this procedure [38].

#### 4.1.6. Sol–Gel Method 

**Figure 5:**
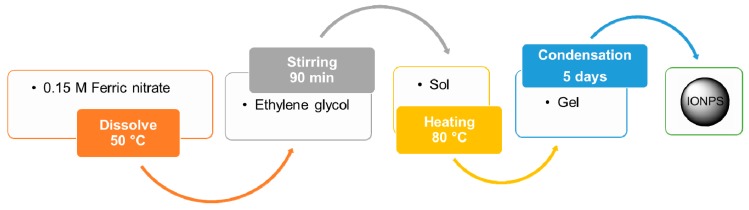
Flowchart for the synthesis of IONPs by sol-gel method [41,42]. Ferric nitrate is directly dissolved in ethylene glycol at 50 °C. The resulting sol is then dried by heating to get the gel, and finally, the IONPs of interest.

This method for IONP production consists in the hydroxylation and condensation of some iron precursors forming a “sol” (i.e., a colloidal solution) of nanoparticles that is then dried—or ‘‘gelled’’—by solvent removal until an iron oxide 3D network is obtained (see Figure 5) [39,40]. In this process, water is usually used as a solvent; alternatively precursors can also be hydrolyzed by acids or bases. The reaction can be performed at room temperature (see Table 1) [41] and the size of the resulting spherical IONPs can be tuned between 15 and 50 nm [42].

#### 4.1.7. Biomineralization

Many living organisms—including humans—have a genetically encoded ability to synthesize minerals and other inorganic substances in a process known as bio-mineralization. In particular, some species are able to produce “biogenic” magnetic nanoparticles on which they base their sense of direction. For example, magnetotactic bacteria can generate magnetosomes (protein-coated nanosized crystals of magnetic iron oxide) [43,44]. For the typical parameters (time, temperature, and MNP size) characterizing these bio-mineralization processes, see Table 1.

#### 4.1.8. Sputter Deposition

A commonly used physical way for IONP synthesis is the so-called sputter deposition. By this technique, Fe atoms are extracted from a solid iron target material (working as a cathode) after its bombardment through energetic particles. Iron atoms sputtered—for instance—by a high-pressure magnetron-sputtering gun are subsequently decelerated into a cooled growth chamber as a result of their collisions with the atoms of Ar and He gas present in the chamber. The so formed iron NPs are then ejected from the aggregation chamber through a pressure gradient system and then deposited onto a sample holder in the adjacent deposition chamber. Afterwards, a small amount of oxygen is introduced in the deposition chamber to allow the oxidation (Fe_3_O_4_) of NP surface, thus leading to the formation of iron/iron-oxide core-shell nanoclusters [45]. By adjusting the physical parameters of the process—such as the pressure in the aggregation chamber, the ratio of He to Ar gas, and the magnetron-sputter power—NP mean diameter can be tuned from 1 to 100 nm [46,47]. Notably, also NP ferromagnetic properties can be changed by ion irradiation [48,49,50].

#### 4.1.9. Application to CNS

As well as an improved biocompatibility and bioavailability, IONPs provide specific advantages for the treatment of neurological disorders based on their possibility to be imaged and externally driven through magnetic scanners for a targeted drug-delivery (theranostic approach). Importantly, external static magnetic fields could also be used to enhance BBB permeability against IONPs, whereas alternating magnetic fields are suitable for selectively killing tumor cells via localized hyperthermia [6]. Among the methods reviewed above, IONPs prepared by co-precipitation, thermal decomposition, microemulsion, and sol-gel, usually exhibit superior targeting capabilities if compared with other synthesis methods [17,22,28]. On the other hand, the main advantage of co-precipitation, hydrothermal and biomineralization synthesis is that they do not require harmful solvents—water being the most used ones—for the preparation of IONPs, thus reducing their toxic potential, especially on neurons [31].

### 4.2. Characterization of IONPs

After the synthesis of IONPs, the characterization of their physicochemical properties can be provided by means of several sophisticated techniques (see Table 2). In the next sections, the focus will be on the techniques used to investigate shape, size, size distribution, and magnetic properties of IONPs.

#### 4.2.1. Microscopic Techniques

Due to the very high resolution achieved by electron microscopy (<1 nm), both Scanning Electron Microscopy (SEM) and Transmission Electron Microscopy (TEM) are widely used to determine, respectively, the surface morphology and the inner structure of NPs. Unlike SEM that produces images making use of reflected (or knocked-off) electrons, TEM indeed works by detecting transmitted electrons that may carry valuable information on the IONP inner structure (e.g., crystal structure, stress state, and more) [57]. Moreover, nanometric investigation of shape heterogeneity, average size, and dispersion can be accomplished by using scanning tunneling microscopy and atomic force microscopy [14].

#### 4.2.2. Spectroscopic Techniques

Many spectroscopic techniques are used for IONPs characterization. X-ray diffraction allows collecting information about the crystalline structure of the particles (i.e., angle, width), while structure characterization and functional group determination, are commonly performed by Fourier-Transform Infrared (FTIR) spectroscopy [58,59], thanks to the presence of molecules absorbing light in the region from 2.5 μm to 15 μm (wavenumber from 4000 cm^−1^ to 660 cm^−1^). Alternatively, NMR (Nuclear Magnetic Resonance) techniques can be used to study the structure of a compound. The ability of NMR to provide information regarding the specific bonding structure and stereochemistry of molecules of pharmaceutical interest has made it a powerful analytical instrument for structural determination [60]. Furthermore, the thermal stability of IONPs can be determined by using the thermogravimetry analysis [61]. Dynamic Light Scattering (DLS) has several advantages for sizing MNPs and it has been widely used to determine the hydrodynamic diameter of IONPs (typically ranging between 30 to 190 nm) via the Stokes-Einstein equation [62]. Moreover, IONPs can be electrostatically characterized by using Zeta potential measurement in order to determine their surface charge (see Table 2). In general, surface charge, and in turn, the Zeta potential can be modified by using polymer coating [63,64]. Mass spectroscopy requires a very small amount of sample to determine molecular weight and surface properties with high accuracy and precision. Fluorescence correlation spectroscopy (using the visible and UV radiation) is normally employed for studying concentration effects, molecular diffusion, and chemical kinetics. Surface-enhanced Raman spectroscopy and circular dichroism make it possible to determine IONP structural conformation; Raman spectroscopy in particular offers the unique possibility to record spectra directly in aqueous solution, without the need for any sophisticated sample preparation [14] (see Table 2).

#### 4.2.3. Magnetometric Techniques

The magnetic properties of IONPs can be assessed by measuring the magnetization of a samples when subjected to an externally applied magnetic field *H* varying from −10,000 Oe to 10,000 Oe, at a defined temperature. The Vibrating Sample Magnetometer (VSM) and Superconducting Quantum Interference Device (SQUID) are the most common techniques able to measure important magnetic parameters of MNPs, such as saturation magnetization, coercive field, and remnant magnetization [65,66]. To date, these measurements represent an essential characterization step, because of the interplay between MNPs and the external magnetic fields employed for magnetic-driven nanocarriers as useful strategies for numerous biomedical applications. In the next section of this review, the magnetic properties of MNPs and their link with the methods of synthesis are investigated in more detail.

#### 4.2.4. Magnetic Properties of IONPs

The MNPs design, particularly related to their physical and chemical properties, can be controlled to fit different biomedical applications, allowing an accurate control of their spatial, temporal, and dosage distribution [1]. The increasing interest in nanomedicine has aroused a special focus on fabrication protocols for high-performance IONPs, for obtaining not only the desired physicochemical properties, such as: Morphology, size, and structure, but also a good biocompatibility [67,68]. 

As reported in Section 4.1, many methods have been developed for IONPs production allowing shape, size and structure controlled synthesis [69,70]. The methods most used in the biomedical field are thermal decomposition of metallic precursors, co-precipitation, and hydrothermal synthesis, in which pressure and reaction temperature values are critical to control the growth and nucleation of IONPs from solution [4]. Many researchers successfully applied these methods for IONPs, in order to obtain nanostructures with various shapes [71,72]. Additionally, efforts have been applied not only for the synthesis of 0-dimensional IONPs, but also of 1 and 3-dimensional structures, developed via a microwave-assisted procedure, also showing that sophisticated morphology (i.e., Fe_3_O_4_ nanotubes or elliptical nanorings) has effective magnetic properties [73,74]. In summary, different morphology and dimensions can be obtained by carefully adjusting the main parameters of the reactions during the synthesis process such as heating temperature, molar ratio, or concentration of the reactants.

We will examine how IONP physical characteristics could impact on magnetic properties.

**Figure 6:**
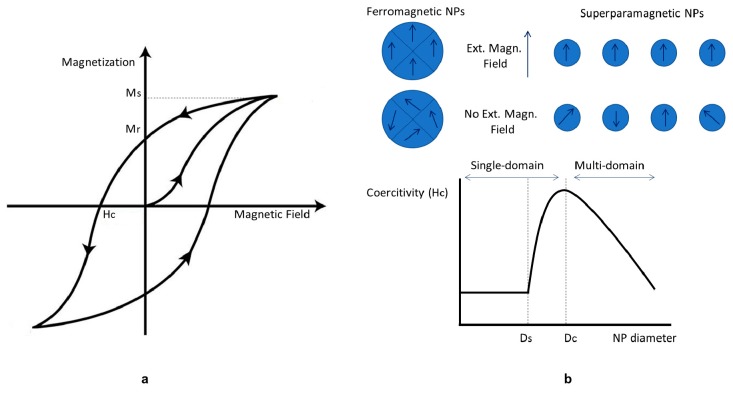
Principal features of magnetic material: (**a**) Hysteresis loop of ferromagnetic and ferrimagnetic materials. Critical magnetic parameters: Saturation magnetization (*M_s_*), Coercive field (*H_c_*), and Remnant Magnetization (*M_r_*) are shown. (**b**) Magnetization behavior of ferromagnetic and superparamagnetic NPs under an external magnetic field. Domains of a ferromagnetic NP align with the applied fields. The magnetic moment of single domain superparamagnetic NPs aligns with the applied field. In the absence of an external field, ferromagnetic NPs will maintain a net magnetization, whereas superparamagnetic NPs will exhibit no net magnetization due to rapid reversal of the magnetic moment. The relationship between NP size, the magnetic domain structures, and coercive field is shown. Ds and Dc are the ‘superparamagnetism’ and ‘critical’ size threshold. Modified from Reference [77].

The various materials can be classified by different form of magnetic behaviors, based on their response to an external magnetic field that, at the microscopic level, interacts with atomic dipoles, causing a measurable macroscopic magnetic moment. Five basic types of magnetism are known: Diamagnetism, paramagnetism, ferromagnetism, antiferromagnetism, and ferrimagnetism. Ferrimagnetic and ferromagnetic materials are the most interesting ones, exhibiting remarkable magnetic properties. In particular, ferromagnetic materials show a high magnetization *M* during the interaction with the applied field *H*. The magnetization does not increase indefinitely, but reaches asymptotically the saturation magnetization *M_s_*. After turning off the external field *H* a little amount of residual magnetization, *M_r_* remains and the applied magnetic field required to reduce to zero the magnetization of the material is called coercive field or coercitivity *H_c_*. This relationship between *H* and *M* is plotted in Figure 6a, showing the magnetic hysteresis loop [75].

The dimension of IONPs, determines the surface-to-volume ratio in turn linked with their reactivity, also responsible for attraction and agglomeration phenomena. In fact, IONPs magnetism is dominated by size effects, due to the magnetic domain structure of ferromagnetic material (i.e., Weiss domain, volume in which all atomic magnetic dipole are aligned). In 1930, Frenkel and Dorfman suggested the principle of the superparamagnetism theory, stating that ferromagnetic materials transfer from multi-domains to a single-domain state by particle resizing to nanoscale (Figure 6b). The existence of a critical size at which the transition to single-domain (i.e., state of uniform magnetization) occurs is well established in the literature. It happens when the magnetic energy configuration of multi-domains is no more favorable compared to the single domain: The gain in energy through the division is less then than the energy of the domain wall. It can be calculated analytically [76] for a sphere or an infinite cylinder as:

*D_c_* = *P* [(*A K_u_*)^1/2^/(*μ*_0_ × *M_s_*^2^)], (1)

where *D_c_* is the critical diameter, *P* is a dimensionless constant depending on the shape (~34 for a cylinder, ~72 for a sphere), *A* is the exchange stiffness constant, *K_u_* is the uniaxial anisotropy constant, *μ*_0_ is the magnetic permeability in vacuum, and *M_s_* is the saturation magnetization [66,77]. For real NPs (1) still approximately holds using a *P* that is generally in the range 20–80.

Decreasing further their size, the IONPs reach the superparamagnetic diameter *D_s_*, showing zero coercivity and no hysteresis, due to the thermal effects. In fact, when the external magnetic field is switched off, the magnetic domains point at random orientation, with zero resultant. The degree of alignment of magnetic moments depends on temperature, it decreases when the temperature increases and vanishes beyond a critical temperature, where the magnetization becomes zero. Above this critical temperature (i.e., blocking temperature *T_b_*), the characteristics of superparamagnets are identical to those of paramagnets with a very high reactivity, i.e., they lose magnetism after the removal of the applied field but maintain an extremely large moment during the interaction. In general, IONPs, with Fe_3_O_4_ as the most magnetic material, can exhibit superparamagnetic properties at room temperature when their size is small enough [66], they are generally called Superparamagnetic Iron Oxides Nanoparticles (SPIONs) or Ultra-Small Iron Oxides nanoparticles (USPIONs), with size >50 nm or <50 nm, respectively [3]. Working with SPIONs in the biomedical field is highly recommended, because of their high magnetization response under external field interaction and their no zero magnetization behavior without applied field, avoiding the risk of agglomeration, and reciprocal attraction [6].

Drawing the magnetization curve (*M-H* curve) for different preparation of IONPs (different size, shape, etc.) is useful to study the link between magnetic and physical properties. This is generally done by means of VSM or SQUID [66]. High *M_s_* is an important parameter for characterizing IONPs, which is typically is measured in emu∙g^−1^, mainly depends on the chemical composition of NPs. Low values of coercive field *H_c_*, measured in Oe, together with the remnant magnetization *M_r_*, are other important indicators of emerging superparamagnetic properties.

**Figure 7:**
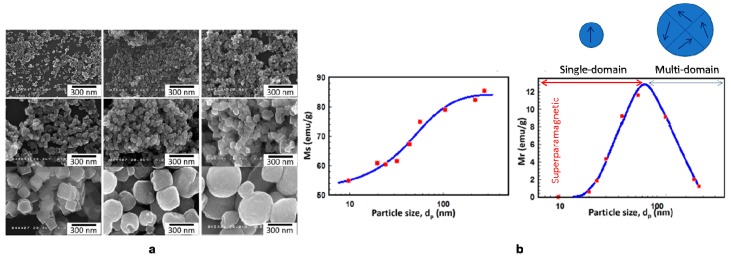
(**a**) Scanning electron microscopy images of cube-like Fe_3_O_4_ nanoparticles with various particle sizes. (**b**) Cube-likeFe_3_O_4_ nanoparticles size dependence of saturation magnetization and remnant magnetization. Red-circles represent cube-like nanoparticles. Modified from Reference [78].

Different critical sizes have been investigated for IONPs, but typically the optimal range for biological applications is considered around 10–100 nm. For instance, Li and colleagues studied the transition from multi to single-domain, establishing the critical size value (~76 nm) for highly crystalline cube-Fe_3_O_4_ NPs. Additionally, the effect of shape was investigated., revealing higher values for *H_c_* and *M_r_* values of cubic NPs with respect to the spherical, due to the different orientation of polycrystalline structure and the consequent spin alignment (Figure 7) [78].

The effect of size on magnetic properties of Fe_3_O_4_ NPs embedded in SiO_2_ matrix was studied by Fuentes-Garcia and colleagues, showing that the coercive force becomes zero for a critical size of about 20 nm [79].

In the last few years, many researchers characterized IONPs by their magnetic profile or compared different materials included in the formulation and structure of IONPs [80,81,82,83]. See Table 3 for an overview of some IONPs synthesis methods, magnetic, and physical properties.

#### 4.2.5. Application to CNS

Focusing on CNS applications, the characterization of IONPs is fundamental to elucidate phenomena such as interaction with magnetic field and agglomeration. In this context, the main uses of IONPs are as a contrast agent for Magnetic Resonance Imaging (MRI), as heating sources in hyperthermia, and as targeted drug-carriers with BBB-crossing capabilities [6]. For example, octapod SPIONs, characterized by TEM analysis and SQUID, showed a more effective MR contrast effect with respect to spherical ones, allowing an accurate detection of tumors [72]. This novel strategy could be useful also for hyperthermia treatment and magnetically guided drug delivery in CNS. Shevtsov and colleagues studied chitosan-dextran SPIONs for the treatment of glioblastoma, the most devastating brain cancer [80]. The accumulation of NPs in the tumor cells was evaluated by means of histological studies and such SPIONs exhibited enhanced properties for the targeted delivery of chemotherapy and for the delineation of the tumor mass in the brain. Moreover, the synthesis strategy described in [79] (IONPs stabilized SiO_2_ matrix) avoided agglomeration phenomena and the characterization by SQUID revealed an effective magnetic excitation to produce hyperthermia.

Finally, the magnetic properties are critical for the passage of IONPs across the endothelial monolayer. Static magnetic fields can facilitate the entry of IONPs into the brain side and the proper delivery of their drug cargo. In addition, alternating magnetic fields can increment BBB permeability by magnetic heating [6]. In this regard, Kaushik et al. produced NPs of CoFe_3_O_4_ for non-invasive magnetically-guided delivery across BBB in mice. The uniform distribution was evaluated using in situ TEM and SEM in ultrathin sections of mouse brain, showing minimal agglomeration [77]. This study provides evidence for the great potentiality of the coordinated use of magnetic forces for nondestructive BBB crossing and the site-specific delivery of therapeutics to treat CNS diseases, such as Alzheimer’s and brain tumors. 

### 4.3. Functionalization of IONPs

**Figure 8:**
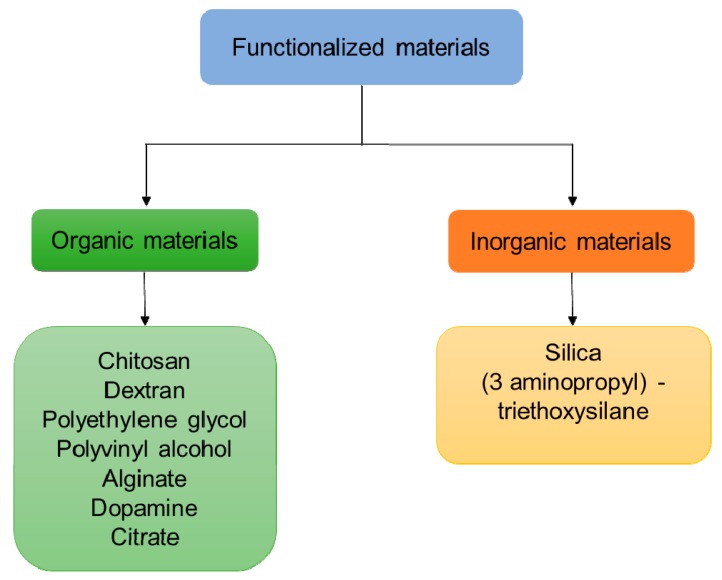
Flowchart of classification of organic and inorganic materials used for the functionalization of IONPs [51].

The most important limitation to drug delivery in the brain is the presence of the BBB, which virtually inhibit the diffusion of the majority of substances to neurons [6]. IONPs can be used to overcome this limitation, but attention has to be posed to possible induced neurotoxicity caused by their oxidation and to mechanisms for enhancing and/or controlling the barrier trespassing [9]. Naked IONPs without coating face many problems such as the aggregation in water, chemical instability in the air, poor biodegradability in the physiological environment, and non-specific interaction with serum protein [86]. To prevent iron oxidation and to minimize the direct exposure of the biological surfaces to IONPs, coating with a biocompatible shell is required. The interaction with cells and functionalized NPs strongly depends on the inorganic or organic materials used [87] for the shell creation (Figure 8). The main purposes of surface modification of IONPs are the following:

1. To improve or modify dispersion in tissues;
2. To improve the surface activity;
3. To enhance physicochemical and mechanical properties;
4. To improve biocompatibility.

An example of the second function, relevant for CNS, is the conjugation with positively charged biomolecules (e.g., peptides or cationic proteins such as albumin) that could facilitate the internalization of IONPs, loaded with drugs, across the BBB [9].

In this section, the main methods of IONP functionalization are summarized, with a particular attention to CNS applications [88].

It is worth mentioning that IONPs can be also used to add magnetic properties to other engineered particles. For example, they have been used to decorate the polymeric shell of nanobubbles [89].

#### 4.3.1. Ligand Addition

**Figure 9:**
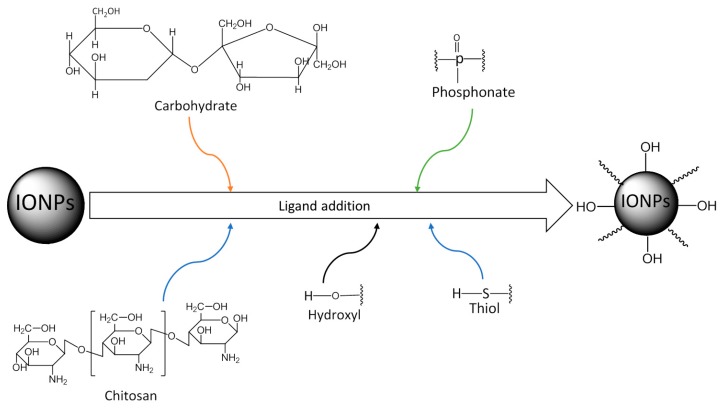
Surface modification of IONPs by ligand addition.

The basic principle of this method is the addition of a ligand molecule to the external surface of the IONPs without eliminating any preexisting ligand. Many functional groups such as hydroxyl, carbohydrate, thyol, and phosphonate can bind to the surface of IONPs (Figure 9) [90].

#### 4.3.2. Ligand Exchange

Ligand exchange is the most commonly used method for controlling the surface properties of IONPs [91]. The hydrophobic nature of the IONPs can be switched to hydrophilic by ligand exchange [92]. In the ligand exchange a new type of functional group replaces the initial hydrocarbon layer directly. The new ligand contains two different kinds of functional groups: 1) Functional groups, which can directly link to the surface of the IONPs tightly by the effect of their chemical bonding; and 2) other functional groups that help particles dissolve in water solution [91].

#### 4.3.3. Silica Coating

Silica is the most used compound for surface coating of IONPs to reduce the toxicity [93]. Silica coating is widely applied to the surface functionalization of NPs and also to improve stability in water and it protects them in an acidic environment [51]. In general, silica coating increases the particle size and modifies the magnetic properties of IONPs. It helps in binding with the various ligands at the surface and provides a protective layer for drug molecules and IONP itself [94]. As listed in Table 4, different methods of silica coating for the IONPs exist. In addition, small molecules such as dyes, drugs, and even quantum dots can be consolidated into the silica shell during its formation. Silica surface can be covalently attached to different ligands and biomolecules to target organs via antibody-antigen recognition [95]. Moreover, silica coating enhances colloidal stability with a relatively easy regulation of the coating process, and as already stated, it can contribute to reduce the IONPs toxicity, a particularly critical aspect for all the CNS applications [96].

#### 4.3.4. Aminosilane Coating 

Aminosilane (AmS) coated IONPs are widley used as a multifunctional drug delivery system. Surface modification of IONPs by AmS can indeed prevent aggregation and allows the addition of specific functionalities to IONPs [97,98]. As for their biocompatibility, it has been shown that AmS-coated particles affected cell metabolic activity only at high concentrations (around 200 μg/mL), while leaving the membrane intact [97]. On the other hand, AmS-IONPs at concentrations above 200 μg/mL have been shown to reduce neuron viability by 50%, both in the presence or absence of a magnetic field [98].

#### 4.3.5. Polymer Coating

Polymer functionalized IONPs have been extensively investigated for their unique physical and chemical properties. The polymer-coated iron oxide nanoparticles (PIONPs) are used in various biomedical applications such as PET imaging or fluorescent imaging (Table 5). Recently, PIONPs have been used to treat anemia and neurological disorder [99]. 

#### 4.3.6. Application to CNS

In recent years, IONPs functionalized by means of different types of coating were investigated for the treatment of important CNS pathologies. The improvement of biocompatibility with respect to naked IONPs is a crucial point of their surface modifications. In this regard, silica-coated SPIONs showed good biocompatibility and an effective magnetic-guided deliverability into the diseased brain regions [94]. Alternatively, the coating of SPIONs by means of organic materials also provided high safety levels, allowing a more extensive use of these agents in the current and future CNS applications. For example, the stability of dextran-SPIONs for MRI application following storage at different temperatures was monitored in Reference [100], showing good stability and the lack of agglomeration or sedimentation. Chitosan is another natural polymer used in various CNS application, such as drug delivery. The main benefit deriving from chitosan coating consists in the support function it can provide for different type of drugs, without impairing the NP properties. Unfortunately, chitosan presents also some limitations due to its reduced solubility in water at physiological pH, thus making chemical changes for improved water-solubility necessary [93]. For this purpose, an interesting hybrid chitosan-dextran SPION system has been shown to be able to enhance the internalization inside glioblastoma cells, revealing how new formulations can improve cellular uptake [80]. Similarly, antibody-conjugated polyethilene glycol-coated IONPs were shown to promote the internalization in brain tumor cells with a high stability degree when suspended in biological media [102]. Ivask and colleagues compared uptake and transcytosis of different functionalized SPIONs in an in vitro BBB model [105]. Overall, these findings point out that polymeric coating is one of the most powerful strategies for improving the internalization of IONPs by the endothelial cells of the BBB and the consequent release in the CNS. In particular, dextran, chitosan, and also polyethylene glycol are the best candidates to study optimized formulations of coated IONPs and improve targeted drug transport across BBB.

### 4.4. Toxicity in CNS

As previously mentioned, several IONPs applications for CNS pathologies have been proposed, but their toxicity for living organism still remains an opened issue. Safety concerns were raised regarding their application, due to contradictory results about neurotoxicity [6].

It is well established in literature the high complexity of mechanisms that can induce cytotoxicity after NPs interaction and involve both cellular and molecular pathways. They include physical damage of membrane, structural changes in cytoskeleton components, defects of transcription and oxidative damage of DNA, damage of mitochondria, disturbance of lysosome functioning, generation of reactive oxygen species (ROS), alteration of membrane protein functions, and synthesis of inflammatory factors and mediators [106]. Nevertheless, a satisfactory understanding of the interactions with biological systems is still missing. Currently, several studies on their harmful effects are carried out in various in vitro and in vivo models, in order to investigate the relation between biocompatibility and NPs or MNPs features [88,93].

Focusing on IONPs, their physicochemical features, such as size, shape, surface charge, and coatings, determine their cytotoxicity. For example, IONPs of few nanometers (approximatively <10 nm) are more toxic than larger ones, which cannot enter the nucleus. The dimension of IONPs is also a significant factor for the biodistribution at body level, affecting the blood circulation time and the filtration from spleen, liver, or kidneys [106]. 

**Figure 10:**
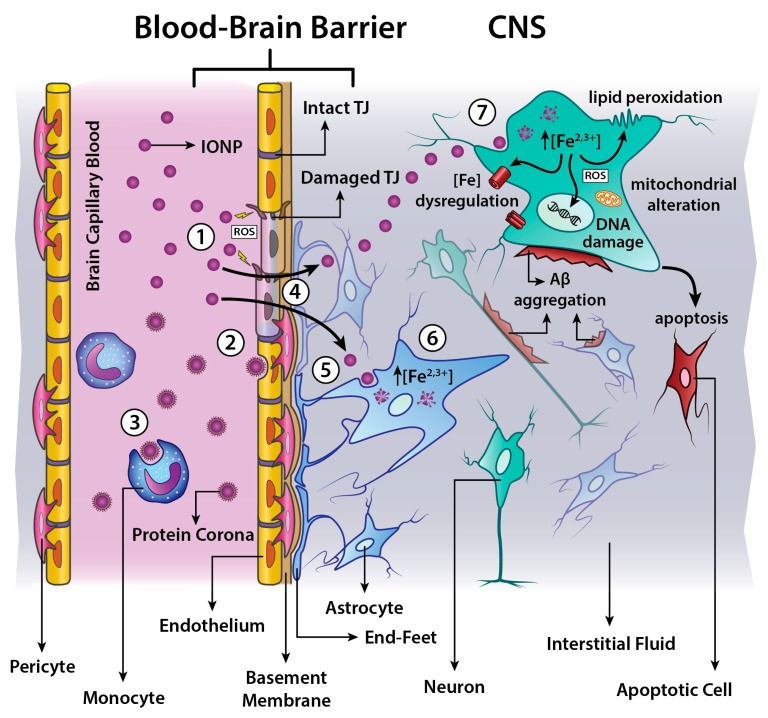
Interference of IONPs with CNS iron homeostasis and its involvement in neurodegeneration process. (1) IONPs in the bloodstream can alter the permeability of BBB. The integrity of BBB can be damaged by ROS production and/or inhibition of Occludin-proteins (belonging to tight junctions proteins TJ); (2) based on their size, surface charge and coating IONPs are subject to the formation of protein corona around IONPs, leading to opsonization phenomena. This can alter physicochemical properties of IONPs and/or improve transport across BBB; (3) IONPs can be also eliminated from blood circulation by means of recognition and uptake by monocytes or other cells of Mononuclear Phagocytic System; (4) IONPs can cross BBB by means of paracellular or transcellular mechanism and enter in CNS [6]; (5) according to their size, IONPs can be internalized by pinocytosis (dimensions < 10 nm) or endocytosis (approximatively larger than 10 nm) into the CNS cells; (6) in the intracellular environment IONPs are degraded in the acid environment of endosomes (degradation time depending on IONP size) with consequent release of iron ions in the cytoplasm; astrocytes are more sensitive to uptake and accumulation of IONPs [111]; (7) in the neurons, after the IONPs internalization and degradation, iron ions participate to redox cycling taking part to many biological processes implicated in neurodegeneration and neural damage: (a) Fenton’s reaction with production of ROS (consequent lipid peroxidation and/or DNA damage); (b) alteration of iron regulation proteins (responsible for iron transport, uptake and storage); (c) alteration of mitochondrial activity; (d) accumulation in iron pool; (e) apoptosis; and (f) protein aggregation (e.g., Aβ, α-synuclein) [108].

In general, IONPs are assumed to be biocompatible, but upon intracellular degradation, IONPs do release iron ions, influencing iron homeostasis at general body level. Especially, due to the high vulnerability of CNS for iron imbalance, much work still has to be done to fully understand how different types of IONPs could affect the brain, BBB (see Figure 10) and what potential adverse effects on CNS can derive from their exposure [12,107]. This is particularly critical in brain neurodegenerative processes as iron homeostasis unbalance impact on the evolution of the diseases or could be involved in the pathogenesis mechanisms [6,108,109]. Yarjanli and colleagues reviewed a large number of studies concluding that IONPs, according to their physicochemical properties, can lead to iron accumulation, oxidative stress, protein aggregation in the neural cells, and may induce neurodegeneration [108]. Additionally, the review of Xie and coworkers evaluated the close links between biocompatibility and size, concentration, surface properties, morphologies and structures of IONPs, underlining the need of carefully engineering biocompatible NPs with in vitro study of degradation and cells availability to ensure in vivo safe metabolization [4].

In vitro experiments aimed at evaluating the effects of IONPs on the CNS, used different cell models (i.e., human brain microvascular endothelial cells, astrocytes, or neurons cell cultures) [107]. The main indicators for toxicity considered by literature are [110]:

- dose and time-dependent cell viability and/or proliferation;
- production of Reactive Oxygen Species (ROS);
- cell membrane disruptions;
- alteration of mitochondrial activity;
- genotoxicity induction.

Several in vitro assessments of IONPs toxicity in CNS investigate the interconnection between NPs physicochemical features and glial and neuronal cells activity. SPIONs made of Fe_2_O_3_ (diameter 51.88 nm) showed neurotoxic effects in PC12 cell line, in a dose-dependent manner at 60–200 μg/mL, but not at 10–50 μg/mL [85]. This is in agreement with a proposed cytotoxicity threshold of 100 μg/mL [4]. Moreover, after short and long-term exposure of human brain cells to IONPs, cytotoxicity and proliferation impairment are proved, also revealing that astrocytes are more vulnerable than neurons [111,112].

Conversely, Dextran-coated SPIONs (10–50 nm) showed to be safer, did not impair the hippocampal cell viability at low doses after 24 h of incubation [113], which confirm the positive effect of the polymeric coating [4,108], as also discussed in Section 4.3. 

The majority of neurotoxicity studies on IONPs are performed in 2-dimensional in vitro cultures [83,85,109,112,113]. There is, however, an increasing interest on co-cultured and 3-dimensional cells cultures, which are more realistic models with respect to 2D systems [114,115], extended also to other types of inorganic NPs, such as silica NPs [116]. For instance, brain cells can be cultured to form spheroids, which allow recreating the 3D-spatial environment of the CNS, in order to test advantageously and more precisely IONPs toxicity. In summary, the exploration of the impact of various IONPs on CNS functions and the development of new strategies to test it are a current research trend running in parallel with the study of iron homeostasis alterations involved in neurodegenerative disorders. 

## 5. Conclusions and Future Perspectives

Due to their unique physicochemical properties, IONPs are considered as one of the most promising tools for biomedical applications because of magnetic driving via external field. In particular, IONPs could be ideal for the physical targeting of CNS, optimizing the crossing of BBB and the drug delivery into the brain. For this reason, the development of engineered IONPs by means of controllable synthesis methods and a careful tuning of their properties are in constant progression.

This review provides an up-to-date overview about the possible strategies to successfully design suitable IONPs able to move from the proof-of-concept level to future clinical settings. The need of an accurate selection of physicochemical properties, such as size, shape, and structure, adjusting the parameters of the synthesis process is the first required step. At the same level, the functionalization of IONPs with different polymers or chemical compound is essential for the improvement of various biomedical applications in CNS. A deeper investigation on the magnetic characterization of IONPs is necessary, in order to better understand the interaction of IONPs with the external magnetic field used as a driving/delivery system. The emergent property of superparamagnetism makes IONPs appealing nanomaterials, as proved by the large number of current studies focused on the detailed analysis of magnetic properties, such as saturation magnetization, coercive field, and remnant magnetization. These parameters are critical for the interplay with the applied magnetic field, allowing the modulation of magnetic-driven nanocarriers in the biological environment.

Furthermore, special attention must be paid to the assessment of biocompatibility and potential risk associated with the IONPs exposure. The low toxicity of IONPs is generally assumed, but since results are often contradictory, further investigation is required, also developing innovative cell models to test it. This is a crucial point due to the high vulnerability of CNS for iron imbalance, especially in the alterations of iron homeostasis strongly connected with neurodegenerative disorders.

In conclusion, a challenging aspect is the need of new smart magnetic nanocarriers able to cross BBB and to reach the brain from bloodstream effectively and safely. For this purpose, the use of an integrated approach taking into account type, composition, functionalization, and minimization of toxicity can promote the optimal choice for magnetic-field-directed NPs.

## Figures and Tables

**Table 1:** IONP synthesis techniques and comparison with respect to their different parameters.

Methods	Temperature	Time	Size	Magnetic Properties	References
Co-precipitation	20–150 °C	minutes	5–40 nm	*M_s_* = 36.8 emu∙g^−1^	[17,18,20,51]
Thermal decomposition	100–350 °C	hours-days	4–30 nm	*M_s_ =* 13.4–49.5 A∙m^2^∙kg^−1^	[22,25,52]
Microemulsion	20–80 °C	hours	10–25 nm	*M_s_ =* 81 emu∙g^−1^ *M_r_ =* 10 emu∙g^−1^ *H_c_ =* 130 Oe	[28,53]
Hydrothermal	150–280 °C	hours-days	10–800 nm	*M_s_ =* 35–40 emu∙g^−1^ *H_c_ =* 0.6–15.7 Oe	[31,32,33,54]
Polyol	130–220 °C	hours	4–100 nm	*M_s_ =* 197–243 emu∙g^−1^	[37,38]
Sol-gel	25–200 °C	hours	15–50 nm	*M_s_ =* 37.5 emu∙g^−1^	[39,55,56]
Biomineralization	80 °C	hours	~140 nm	*M_s_ =* 92–100 A∙m^2^∙kg^−1^	[43,44]
Sputter deposition	100–800 °C	hours	5–100 nm	*M_s_ =* 48–71 emu∙g^−1^ *M_r_ =* 2.5–5 emu∙g^−1^ *H_c_ =* 160–220 Oe	[45,46,47,48,49,50]

**Table 2:** Characterization techniques used to evaluate physicochemical properties of IONPs.

Techniques	Evaluation
Infrared spectroscopy (IR)	Nature of surface functionalization
Nuclear magnetic resonance spectroscopy (NMR)	Longitudinal and transverse relaxivity; Structure conformation
Superconducting quantum interference device (SQUID); Vibrating sample magnetometry (VSM)	Magnetic properties
Electron microscopy (transmission, TEM; scanning, SEM)	Morphology, crystallinity, size distribution, composition
X-ray diffraction (XRD)	Crystal structure, size
Dynamic light scattering (DLS)	Hydrodynamic diameter
Zeta potential measurement	Surface charge
Thermal analysis (differential scanning calorimetry, thermogravimetric analysis, etc.)	Surface coverage, thermal stability, nature of surface functionalization, carrier-drug interaction
Mass spectroscopy	Molecular weight
Fluorescence correlation spectroscopy	Dimension, binding kinetics of hydrodynamic
Surface-enhanced Raman scattering	Size distribution, electronic characteristics
Circular dichroism	Thermal constancy
Scanning tunnelling microscopy; Small-angle X-ray scattering	Shape heterogeneity, size and size navigation
Atomic force microscopy	Shape heterogeneity

**Table 3:** Examples of different types of IONPs.

Type of IONPs	Synthesis Method	Physicochemical Properties	Magnetometric Properties	Reference
Fe_3_O_4_	Thermal decomposition of iron oleate in NaCl	Octapodes; 20–30 nm	*M_s_* = 51–71 emu∙g^−1^ *T_b_* = 240–290 K (SQUID)	[72]
Fe_3_O_4_	Liquid precipitation + controlled crystal growth	Crystallite Cube; 10–300 nm	*M_s_* = 54.7–84.7 emu∙g^−1^ *D_c_* = 76 nm (SQUID)	[78]
Fe_3_O_4_ in SiO_2_ matrix	Co-precipitation + Stober modification	Cubic inverse spinel structure; 6–17 nm	*M_s_* = 0.001–0.0015 emu *H_c_* = 24–26 Oe (SQUID)	[79]
Fe_3_O_4_	Thermal decomposition of iron oleate	Cuboid shape; 7–11 nm	*M_s 5 K_* = 11.96 emu∙g^−1^ *M_s 310 K_* = 11.01 emu∙g^−1^ *T_b_* = 181 K *H_c_* = 375 Oe (5 K) (VSM)	[84]
Fe_m_O_n_	Precipitation method	Spherical shape; 28.8–68.1 nm	*M_s_* = 43.8 emu∙g^−1^ (VSM)	[85]
Fe_m_O_n_-SiO_2_	Co-precipitation	Irregular nanoflakes; 98–101 nm	*M_s_* = 11 emu∙g^−1^ (VSM)	[82]
SiO_2_-Fe_m_O_n_	Co-precipitation	Core-shell structure; 98–101 nm	*M_s_ =* 37.2 emu∙g^−1^ (VSM)	[82]

*M_s_* = saturation magnetization; *T_b_* = blocking temperature; *D_c_* = critical size; and *H_c_* = coercivity field.

**Table 4:** Synthesis methods for silica-coated IONPs.

Synthesis Method	Advantage	Disadvantage
**Stöber method**	Controllable silica shell and uniform size, high crystallinity	Lack of understanding of its kinetics and mechanism
**Microemulsion**	Control of particle size	Poor yield, time consuming
**Aeresol pyrolysis**	Hermitically coated	Complex experimental condition

Adapted from [11].

**Table 5:** Common polymers used for functionalized IONPs in biomedical applications.

Polymers	Advantage	Application	Reference
Chitosan	Biocompatible, hydrophilic	MRI contrast agent, Drug delivery agent	[93]
Dextran	Enhance blood circulation, stabilizes colloidal suspension	MRI contrast agent, Molecular diagnostic agent	[100]
Gelatin	Gelling agent, biocompatible emulsifier	Magnetic resonance imaging	[101]
Polyethylene glycol	Internalization efficiency of the NPs	Magnetic hyperthermia agent, MRI theranostics	[102,103]
Polyvinyl alcohol	Prevents particles coagulation	Drug delivery agents, cytotoxicity studies	[104]

## Abbreviations

2D	2-Dimension
3D	3-Dimension
AmS	AminoSilane
Aβ	Amyloid-beta
BBB	Blood-Brain-Barrier
CNS	Central Nervous System
CTAB	cetyltrimethylammonium bromide
DLS	Dynamic Light Scattering
DNA	DeoxyriboNucleic Acid
FTIR	Fourier-Transform Infrared
IONP	Iron Oxides Nanoparticle
IR	Infrared
MNP	Magnetic Nanoparticle
MR	Magnetic Resonance
MRI	Magnetic Resonance Imaging
NMR	Nuclear Magnetic Resonance
NP	Nanoparticles
PET	Positron Emission Tomography
PIONP	Polymer-coated Iron Oxides Nanoparticle
ROS	Reactive Oxygen Species
SEM	Scanning Electron Microscopy
SPIONs	Superparamagnetic Iron Oxides Nanoparticles
SQUID	Superconducting Quantum Interference Device
TEM	Transmission Electron Microscopy
USPION	Ultra-Small Iron Oxides Nanoparticle
VSM	Vibrating Sample Magnetometer
W/O	Water-in-Oil
XRD	X-Ray Diffraction
